# Designing a broad-spectrum multi-epitope vaccine against influenza A and *Mycoplasma pneumoniae*: an immunoinformatics approach

**DOI:** 10.3389/fpubh.2026.1671035

**Published:** 2026-01-21

**Authors:** Zhengyu Yang, Yang Li, Lingling Chen, Shulei Jia

**Affiliations:** 1 Intellectual Property Research Institute, Xiamen University, Xiamen, Fujian, China; 2 National Engineering Research Center for Beijing Biochip Technology (CapitalBio), Beijing, China; 3 School of Basic Medical Sciences, Tianjin Medical University, Tianjin, China

**Keywords:** influenza A, *Mycoplasma pneumoniae*, multi-epitope vaccine, immunoinformatics, dual-target vaccine

## Abstract

**Introduction:**

Influenza A and *Mycoplasma pneumoniae* (*M. pneumoniae*) are common respiratory pathogens, causing severe co-infections in clinical diseases. Current vaccines have failed to provide comprehensive protection against both of the pathogens, highlighting the requirement of integrated solution.

**Methods:**

In this study, a novel dual-target multi-epitope vaccine was developed based on the immunoinformatics method. Based on the consistent sequences, we used the HA and NA proteins of influenza A virus, and the p1, p65, HMW1-3 proteins of *M. pneumoniae* to determine the immunodominant T- and B-cell epitopes.

**Results and Discussion:**

The designed vaccine included 21 linear B-cell epitopes, 34 CTL epitopes and 19 HTL epitopes from influenza A virus and *M. pneumoniae*. The selection was based on antigenicity, immunogenicity, and alignment with previously validated epitopes. Structural and physicochemical assessments indicated that the vaccine had high stability, solubility, and minimal allergy risk. Molecular docking with TLR3 and TLR4 receptors revealed strong binding, suggesting robust immune activation. Population coverage analysis showed the vaccine could cover 97.07% of the global population, with high efficacy across key regions such as North America and East Asia. In this study, we provided an immunoinformatics approach for vaccine design.

## 1 Introduction

The influenza A virus and *M. pneumoniae* are formidable adversaries in respiratory disease. Influenza A virus belongs to the most studied virus and its mutant initiates epidemic and pandemics outbreaks. The high transmissibility and antigenic variability of influenza A virus drive the annual epidemic, with H1N1, H3N2, and avian H5N1 strains alone causing 290,000–650,000 global deaths annually (1). Severe complications, such as acute respiratory distress syndrome, myocarditis, and secondary bacterial pneumonia, arise disproportionately in vulnerable populations, including the older adults and immunocompromised (2). Meanwhile, *M. pneumoniae*, though often dismissed as atypical, accounts for 30–50% of pediatric pneumonia hospitalizations (3), with chronic sequelae such as asthma exacerbations and bronchiolitis obliterans linked to repeated infections (4).

The intersection of these pathogens creates a clinical crisis. Co-infections are not rare; they occur in 12–25% of severe pneumonia cases and lead to a dramatic increase in patient mortality, which can be up to 3.8 times higher than in mono-infections (5–8). The synergistic pathogenesis of co-infection stems from the ability of influenza viruses to disrupt the epithelial barrier of the respiratory tract, exposing receptors like fibronectin that *M. pneumoniae* exploits via its p1 adhesin to establish infection (3, 9). At the same time, *M. pneumoniae* downregulates host immune responses, including interferon-*γ* and IL-12, further facilitating viral replication (10, 11). Clinical outcomes of such coinfections can be devastating, particularly in children and immunocompromised patients. A recent pediatric case study reported fatal outcomes due to combined H1N1 and *M. pneumoniae* infection (12), emphasizing the lethality of this synergy. These findings reinforce the notion that dual infections not only increase symptom severity but also hinder timely therapeutic intervention due to overlapping clinical presentations and diagnostic uncertainty (13, 14). Current vaccines fail to address this syndemic threat. Seasonal influenza vaccines, which target mutable hemagglutinin (HA) and neuraminidase (NA) proteins, offer only 35–50% efficacy in a typical season due to rapid antigenic drift (12). These vaccines do not confer any immunity against *M. pneumoniae*, leaving a substantial immunological gap. The *M. pneumoniae* vaccine development has been difficult due to autoimmune responses in early trials (15). Historical whole-cell formulations led to adverse autoimmune responses (15), stalling progress for decades. This absence of a dual-protective immunization strategy continues to expose millions to preventable morbidity and mortality (8, 15).

To prevent the problems caused by the rapid variability of pathogens, a multi-epitope vaccine consisting of potential T-cell and B-cell epitopes can be an effective approach for preventing the co-infection of influenza A and *M. pneumoniae*. The designed vaccine could produce both cellular and humoral immune response against specific pathogens without producing any immune complications. In this study, to achieve broad-spectrum protection, consensus sequences for key antigenic proteins such as HA, NA, HMW1-3, p1 and p65 were generated from multiple strains of influenza A and *M. pneumoniae*, respectively (12, 15). Then the vaccine was constructed based on selected epitopes from conserved regions of these proteins. This strategy ensures the inclusion of immunodominant epitopes with broad population coverage, strong antigenicity, and high immunogenicity, overcoming the challenges posed by viral variability. The design leverages the antigenic properties of both pathogens and focuses on achieving cross-protection across the influenza A and *M. pneumoniae* variants. We utilized structural biology tools and computational simulations to refine the vaccine, ensuring it would elicit robust immune responses while maintaining stability and solubility for practical use. This dual-target vaccine has the potential to prevent both influenza A and *M. pneumoniae* infections, offering an effective strategy for combating these widespread respiratory diseases.

## 2 Materials and methods

### 2.1 Consensus sequences for structural proteins

To design a multi-epitope vaccine, we selected specific structural proteins from influenza A (HA, NA for strains H1N1, H3N2, H5N1) and *M. pneumoniae* (HMW1-3, p1, p65 adhesion proteins) as antigenic targets. The sequences of these proteins were retrieved from ViPR^1^, NCBI^2^ and the Influenza Virus Database^3^. Following sequence alignment using MAFFT v7.505, a Python script was employed to generate consensus sequences for each protein by retaining the most frequent sites across their sequences.

### 2.2 Forecasting and evaluation of the MHC I and II epitopes

MHC I (9-mer) and MHC II (15-mer) epitopes were predicted using NetMHCpan-4.1 and NetMHCIIpan-4.0 tools (16). The epitopes with the strongest HLA binding degree (<0.5%) were selected for MHC I, while those with a relatively weak binding degree (<10%) were selected for MHC II (17).

The antigenicity of proteins was assessed with the VaxiJen v2.0 server^4^ (antigen score > 0.4) (18). The toxicity was evaluated by using ToxinPred^5^ (SVM-based forecasting approach, *E*-value cut-off: 10) (19). The conservation was analyzed via Epitope Conservancy Analysis^6^.

### 2.3 Immunogenicity, cytokine and population coverage of peptides

The immunogenicity of cytotoxic T lymphocyte (CTL) peptides was predicted using the Class I Immunogenicity tool^7^ (epitopes with score above 0.12). For helper T cell (HTL) peptides, potential induction of IFN-*γ*, IL-4, and IL-10 was assessed by using the IFNepitope, IL4pred, and IL10pred servers. Epitopes and their allelic data were analyzed for population coverage with IEDB (Immune Epitope Database)^8^. Finally, for CTL epitopes, we selected those with high immunogenicity and antigen scores without allergenicity. As to HTL epitopes, we selected those with high IFN-γ and antigen scores with no allergenicity.

### 2.4 Prediction of linear B-lymphocyte epitopes

B-cell epitopes are essential to induce humoral or antibody-mediated immunity. Therefore, we predicted the linear B-lymphocyte (LBL) epitopes using ABCpred server^9^. The ABCpred server utilized an ANN to identify 16-residue B-cell epitopes with an accuracy of 65.93% (threshold: 0.51). Those epitopes with the score over 0.9 were selected for further analysis. The predicted LBL epitopes were also evaluated using VaxiJen v2.0, Immunogenicityserver, ToxinPred, and the AllerTop v2.0 server (17). Finally, those epitopes with high antigen and immunogenicity scores and no allergenicity were selected as eligible linear B cell epitopes.

### 2.5 The multi-epitope vaccine construction

Based on predicted epitopes with optimal antigenicity, immunogenicity, and non-toxic profiles, a multi-epitope vaccine targeting both influenza A and *M. pneumoniae* had been constructed. The influenza A CTL epitopes and HTL epitopes from HA and NA proteins, designed for MHC class I and MHC class II presentation, respectively, were linked with GPGPG linkers to enhance stability and processing. Meanwhile, the B-cell epitopes were linked with the CTL epitopes via the AAY linkers. Similarly, for *M. pneumoniae*, CTL and HTL epitopes from p1 adhesin and HMW1-3 proteins were linked in a similar manner. The AAY linker is a type of cleavage site of proteasomes that was used to influence protein stability, reduce less immunogenicity and enhance epitope presentation. The GPGPG linker is known as the glycine-proline linker, preventing the formation of junctional epitopes and facilitating the immune process. To enhance immunogenicity, the 50S ribosomal protein L7/L12 adjuvant (UniProtKB/Swiss-Prot: P9WHE3.1) was incorporated at the N-terminus via the EAAAK rigid linker (17). Meanwhile, a 6 × His tag was added at the C-terminus for efficient purification.

### 2.6 Assessment of the constructed vaccine

The physicochemical properties and solubility of the vaccine were assessed for performance. These were analyzed using the Expasy Protparam^10^, with solubility predicted by using the Protein-Sol server^11^.

### 2.7 Secondary structure forecasting

The 2D structural features of the vaccine, including alpha helices, random coils, and beta-turns, were predicted using the PSIPRED v4.0 server with default parameters^12^ (17).

### 2.8 Homology modeling, 3D structure optimization and verification

The vaccine’s 3D structure was predicted with the AlphaFold 3.0^13^ and further refined by using GalaxyRefine^14^. The optimal structure was selected based on energy score and RMSD value. Visualization was done with PyMOL v2.5.7^15^, and model quality was confirmed through ProSA-web^16^ and PROCHECK server^17^ (17, 20).

### 2.9 Disulfide engineering of the designed vaccine

The disulfide bonds usually play a crucial part in stabilizing protein folded state by reducing its structural disorderliness and enhancing its energetic stability. In order to enhance stability, the Disulfide by the Design 2.0 website^18^ was used to predict the disulfide bonds of the constructed vaccine. The structure was used to scrutinize potential cysteine mutations, which resulted in the creation of disulfide bonds within the vaccine. The energy of residue pairs ≤ 2.2 kcal/mol was selected as the threshold (17).

### 2.10 Molecular docking

Molecular docking was performed to study the binding of model proteins with receptors. The model of toll-like receptor 3 (TLR3) (PDB ID: 1ZIW) and toll-like receptor 4 (TLR4) (PDB ID: 4G8A) receptors were retrieved from the RCSB Protein Data Bank^19^. They were selected as immunological receptors due to their recognition of viral glycoproteins and double-stranded RNA (dsRNA), which could efficiently trigger robust Th1-biased and cytotoxic T-cell responses crucial for antiviral immunity. The refined model was then docked as a ligand using the ClusPro v2.0^20^. The calculations were carried out with default parameters. Briefly, this involved rigid-body docking followed by scoring of the generated poses with a balanced energy function that accounts for electrostatic and hydrophobic interactions along with a desolvation penalty. The top 1,000 lowest energy structures were subsequently clustered using a root-mean-square deviation (RMSD) criterion, and the central member of the most populous clusters was selected for further analysis.

### 2.11 Normal mode analysis (NMA)

The iMODS tool^21^ (21) was used for normal mode analysis to examine the stability and physical movements of the vaccine-receptor docked complexes. This tool can use NMA in the inner coordinates to predict the collective motions of proteins. The IMODS tool calculates the deformability, eigenvalues, variance, covariance plot, B-factor, and elastic network of vaccine-receptor complexes.

### 2.12 *In silico* vaccine cloning

The JCat website^22^ was utilized for codon optimization, enabling high-level expression in specific host expression systems. We chose *Escherichia coli* K12 as the organism of host expression. We inferred the protein expression level through the CAI (codon adaptation index) and the GC content percentage. The CAI value closer to 1.0 is usually considered optimal, with scores exceeding 0.8 generally regarded as favorable. Meanwhile, the GC content is typically targeted within a range of 30–70% (17, 22).

## 3 Results

### 3.1 Consensus sequence and multi-epitopes prediction

**Figure 1:**
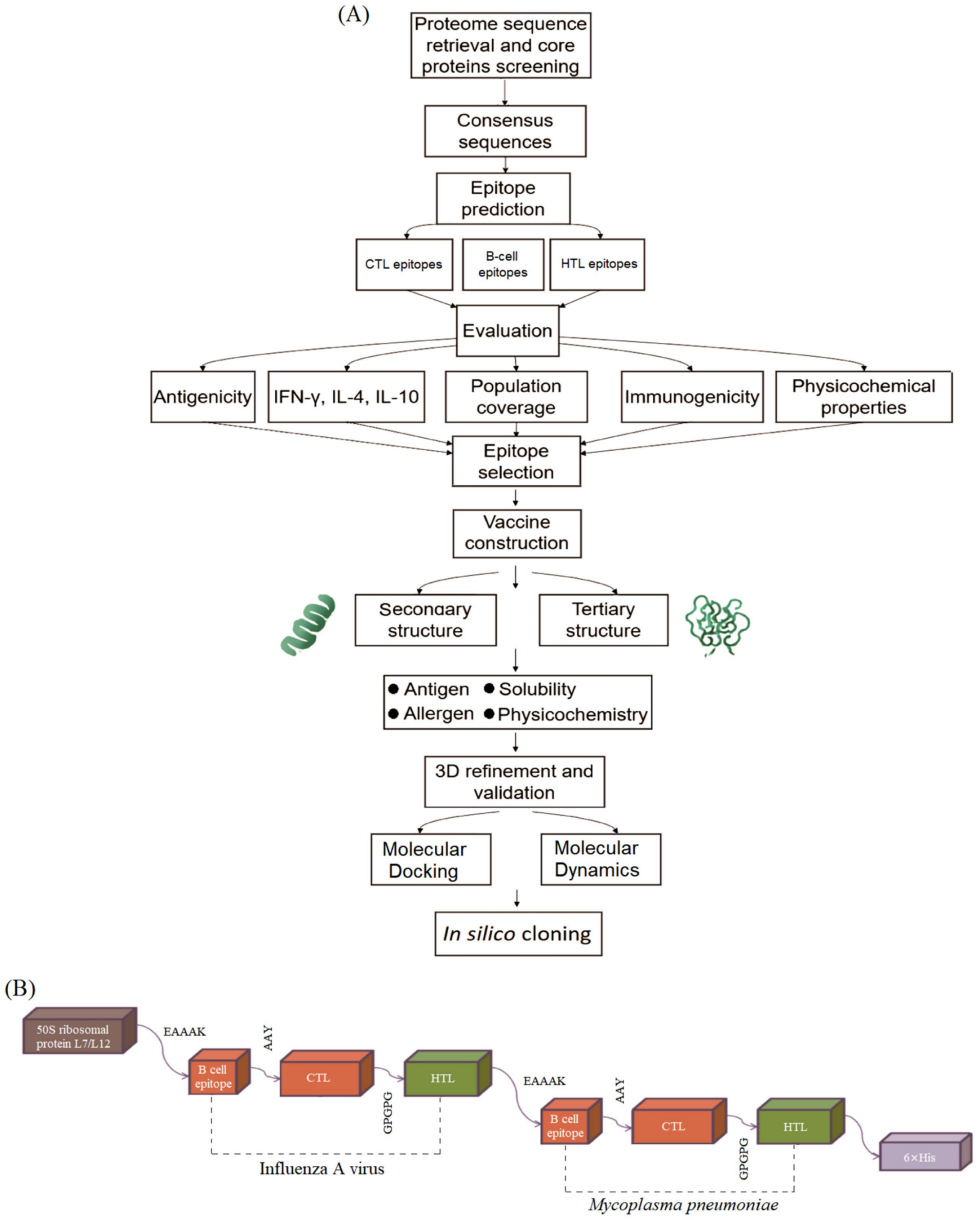
Pipeline for the construction and design of the multi-epitope vaccine. **(A)** The overall workflow illustrates the key stages of vaccine design, from antigen selection and consensus sequence generation to epitope prediction, vaccine assembly, and *in silico* validation of its physicochemical and immunological properties. **(B)** Schematic representation of the final vaccine construct, showing the arrangement of the N-terminal L7/L12 adjuvant, CTL epitopes (linked by AAY), HTL epitopes (linked by GPGPG), B-cell epitopes, and the C-terminal 6 × His tag. This design ensures optimal epitope presentation and facilitates protein purification.

The process of constructing the vaccine involved several steps, starting with the alignment of macromolecular sequences from influenza A (H1N1, H3N2, H5N1) and *M. pneumoniae* to generate consensus sequences. These sequences included the HA and NA proteins from influenza A, and the p1, p65 and HMW1-3 proteins from *M. pneumoniae*. While the HA protein demonstrated greater conservation, the p1 protein exhibited more frequent mutation hotspots (Figures 1A,B).

Based on the antigenicity, immunogenicity and IFN-*γ* scores, 21 linear B-lymphocyte epitopes were selected for further analysis, with 10 from antigens of influenza A and 11 from antigens of *M. pneumoniae.* Meanwhile, 34 cytotoxic T lymphocyte (CTL) epitopes were selected for the final vaccine design. Of these, 10 epitopes were derived from influenza A (HA and NA) and 24 from *M. pneumoniae* (HMW1-3, p1, and p65). Notably, 8 of these epitopes matched those previously validated in the IEDB database, confirming their relevance for inclusion. Additionally, 19 helper T lymphocyte (HTL) epitopes, each 15 amino acids long, were identified from both influenza A and *M. pneumoniae* proteins (Tables 1, 2). All of the epitopes have been manually checked to avoid epitope redundancy or overlap.

**Table 1:** The HTL and CTL epitopes of influenza A selected to construct the candidate vaccine (%percentile rank: MHC I <0.5%, MHC II <10%).

Protein	Types	Grand average of hydropathicity (GRAVY)	Peptide sequence	Length	Location	Alleles	Matched with IEBD	Antigenicity score	Class I immunogenicity	IFN-γ score	IL-4 prediction	IL-10 prediction	Population coverage
B-cell epitopes													
HA	H1N1	−0.050	LVEPGDKITFEATGNL	16	254–269	HLA-A*02:01	–	0.548	0.344	–	–	–	–
		0.863	VPSIQSRGLFGAIAGF	16	342–357	HLA-B*07:02	–	0.565	0.242	-	-	-	–
		−1.094	GWTGMVDGWYGYHHQN	16	361–376	HLA-A*02:01	–	0.509	0.202	–	–	–	–
	H3N2	−1.094	GWEGMVDGWYGFRHQN	16	362–377	HLA-A*02:01	–	0.602	0.358	–	–	–	–
NA	H1N1	−0.188	IGYICSGVFGDNPRPN	16	318–333	HLA-A*24:02 HLA-B*07:02	–	0.473	0.181	–	–	–	–
		−1.113	FEMIWDPNGWTGTDNK	16	375–390	HLA-A*02:01	–	0.44	0.668	–	–	–	–
	H3N2	−0.456	GGDIWVTREPYVSCDP	16	115–130	HLA-A*02:01	–	0.433	0.420	–	–	–	–
	H5N1	−0.831	YHYEECSCYPDAGEIT	16	258–273	HLA-A*03:01 HLA-A*11:01	–	0.55	0.17	–	–	–	–
		−0.588	AGEITCVCRDNWHGSN	16	269–284	HLA-A*02:01	–	0.84	0.383	–	–	–	–
		−0.188	IGYICSGVFGDNPRPN	16	298–313	HLA-A*24:02 HLA-B*07:02	–	0.473	0.181	–	–	–	–
CTL epitopes													
HA	H1N1	−0.767	GRMNYYWTL	9	242–250	HLA-B*27:05, HLA-B*39:01	90%	1.463	0.139	–	–	–	7.46%
		0.022	NIHPITIGK	9	311–319	HLA-A*03:01	–	2.199	0.298	–	–	–	16.81%
	H3N2	1.122	ISIYWTIVK	9	246–254	HLA-A*11:01	100%	0.852	0.428	–	–	–	15.53%
		0.211	GYKDWILWI	9	526–534	HLA-A*24:02	100%	0.465	0.413	–	–	–	21.38%
	H5N1	−0.011	MPFHNIHPL	9	305–313	HLA-B*07:02, HLA-B*08:01, HLA-B*39:01	75%	1.263	0.210	–	–	–	25.02%
		−0.489	KMNTQFEAV	9	404–412	HLA-A*02:01	100%	0.911	0.142	–	–	–	39.08%
		−0.233	KMEDGFLDV	9	429–437	HLA-A*02:01	100%	0.825	0.202	–	–	–	39.08%
		−0.222	VLMENERTL	9	446–454	HLA-A*02:01	100%	0.607	0.198	–	–	–	39.08%
NA	H3N2	0.678	ITTVTLHFK	9	30–38	HLA-A*11:01	–	1.987	0.177	–	–	–	15.53%
		0.267	NELGVPFHL	9	161–169	HLA-B*39:01, HLA-B*40:01	100%	0.590	0.178	–	–	–	10.45%
HTL epitopes													
HA	H1N1	0.553	LCKLRGVAPLHLGKC	15	58–72	HLA-DRB1*01:01, HLA-DRB1*09:01, HLA-DRB1*07:01, HLA-DRB1*15:01	75%	0.803	–	0.551	IL4-inducer	IL10 inducer	48.79%
		−1.133	KKFKPEIAIRPKVRD	15	225–239	HLA-DRB1*01:01, HLA-DRB1*04:01	100%	1.624	–	0.625	IL4-inducer	IL10 inducer	22.06%
	H3N2	−0.693	SRPRIRDIPSRISIY	15	235–249	HLA-DRB1*03:01, HLA-DRB1*15:01	–	0.692	–	0.823	IL4-inducer	IL10 inducer	34.44%
		−0.62	IAPRGYFKIRSGKSS	15	268–282	HLA-DRB1*01:01, HLA-DRB1*04:01	75%	1.046	–	0.546	IL4-inducer	IL10 inducer	22.06%
	H5N1	0.127	FFWTILKPNDAINFE	15	244–258	HLA-DRB1*04:01, HLA-DRB1*09:01, HLA-DRB1*07:01	88%	0.688	–	0.682	IL4-inducer	IL10 inducer	33.74%
		−0.12	ELLVLMENERTLDFH	15	443–457	HLA-DRB1*03:01, HLA-DRB1*04:01	94%	1.045	–	0.973	IL4-inducer	IL10 inducer	27.97%
NA	H1N1	0.173	GVKGFSFKYGNGVWI	15	345–359	HLA-DRB1*07:01, HLA-DRB1*09:01	–	0.830	–	0.465	IL4-inducer	IL10 inducer	24.01%
	H3N2	−0.033	NITEIVYLTNTTIEK	15	61–75	HLA-DRB1*01:01, HLA-DRB1*04:01, HLA-DRB1*15:01	–	0.792	–	0.389	IL4-inducer	IL10 inducer	38.20%
		−0.153	GFAPFSKDNSIRLSA	15	96–110	HLA-DRB1*03:01, HLA-DRB1*04:01	88%	0.535	–	0.671	IL4-inducer	IL10 inducer	27.97%
	H5N1	−0.007	PISNTNFLTEKAVAS	15	48–62	HLA-DRB1*04:01	–	0.407	–	1.213	IL4-inducer	IL10 inducer	11.21%

–: not detected.

**Table 2:** The HTL and CTL epitopes of *M. pneumoniae* selected to construct the candidate vaccine (%percentile rank: MHC I <0.5%, MHC II <10%).

**Protein**	**Grand average of hydropathicity (GRAVY)**	**Peptide sequence**	**Length**	**Location**	**Alleles**	**Antigenicity score**	**Class I immunogenicity**	**IFN-γ score**	**IL-4 prediction**	**IL-10 prediction**	**Population coverage**
B-cell epitopes											
HMW1	−0.544	DTEYDISVLFDANGNP	16	72–87	HLA-DRB1*01:01	0.534	0.241	–	–	–	–
	−0.619	PQAAPQPAVYEWNLTP	16	480–495	HLA-DRB1*04:01	1.016	0.313	–	–	–	–
HMW2	−1.25	QAEITRLKTRNADLEK	16	1,590–1,605	HLA-DRB1*15:01	0.642	0.224	–	–	–	–
HMW3	−0.031	PLSGEGYPDIDAGLPV	16	149–164	HLA-DRB1*07:01	0.522	0.323	–	–	–	–
	−0.256	TPTVEPTPTPVVETAP	16	310–325	HLA-DRB1*07:01	0.862	0.425	–	–	–	–
	−0.15	PKVVEPTPTPVVEATP	16	330–345	HLA-DRB1*07:01	0.81	0.426	–	–	–	–
	−1.175	VQPIIRPTQPEPEWKP	16	466–481	HLA-DRB1*04:01	1.102	0.456	–	–	–	–
	0.212	SGAITIHTTNRSLLLE	16	501–516	HLA-DRB1*15:01	0.679	0.278	–	–	–	–
p1	−1.1	TNAINPRLTPWTYRNT	16	60–75	HLA-DRB1*04:01	1.318	0.486	–	–	–	–
	−0.675	HHGLWDWKARDVLLQT	16	420–435	HLA-DRB1*01:01	1.401	0.306	–	–	–	–
	0.319	PGLAWTPQDVGNLVVS	16	1,101–1,116	HLA-DRB1*07:01	1.018	0.303	–	–	–	–
CTL epitopes											
HMW1	−0.044	SLDPIGETA	9	256–265	HLA-A*02:01	0.872	0.265	–	–	–	39.08%
	−0.244	LQPEPVTEV	9	290–299	HLA-A*02:01	1.139	0.216	–	–	–	39.08%
	0.556	TIAEITPQV	9	326–335	HLA-A*26:01	0.983	0.203	–	–	–	5.82%
	0.722	APPLFEIEL	9	571–580	HLA-B*07:02	1.102	0.364	–	–	–	12.78%
	0.222	AINFDDIFK	9	746–755	HLA-A*11:01	0.468	0.339	–	–	–	15.53%
	−1.022	KLDDFDFET	9	982–991	HLA-A*02:01	1.812	0.322	–	–	–	39.08%
HMW2	−0.9	SRYANWADF	9	133–142	HLA-B*27:05	1.886	0.286	–	–	–	4.78%
	−1.256	KRREIDDLL	9	421–430	HLA-B*27:05	1.128	0.28	–	–	–	4.78%
	−1.078	YQADFENEI	9	527–536	HLA-A*02:01, HLA-B*39:01	0.487	0.296	–	–	–	40.75%
	−0.1	FLEGEFNHL	9	590–599	HLA-A*02:01	0.607	0.288	–	–	–	39.08%
	0.033	KLAERELAI	9	686–695	HLA-A*02:01	1.034	0.272	–	–	–	39.08%
	−0.367	ASKERILDF	9	738–747	HLA-B*08:01	1.08	0.21	–	–	–	10.55%
	0.311	TEELEAAFL	9	836–845	HLA-B*40:01	0.762	0.257	–	–	–	7.81%
	0.844	ELKIAFADL	9	919–928	HLA-B*08:01	1.921	0.258	–	–	–	10.55%
	−0.456	EVLEIENYY	9	1,423–1,432	HLA-A*26:01	0.875	0.314	–	–	–	5.82%
	−1.433	NLAEREREI	9	1,539–1,548	HLA-B*08:01	1.428	0.36	–	–	–	10.55%
	−0.9	YPYPYPWFY	9	1,622–1,631	HLA-A*01:01	0.947	0.229	–	–	–	17.34%
	−1.433	RYENELTEL	9	1,656–1,665	HLA-A*24:02	0.608	0.204	–	–	–	21.38%
HMW3	0.1	SYPDINVVY	9	23–32	HLA-A*24:02	1.364	0.201	–	–	–	21.38%
p1	−0.456	STTFALRRY	9	123–132	HLA-A*01:01, HLA-A*26:01	0.467	0.232	–	–	–	22.62%
	−1.511	TPKWNHHGL	9	414–423	HLA-B*07:02, HLA-B*08:01	0.843	0.224	–	–	–	22.61%
	−2.278	NPRRHPEWF	9	439–448	HLA-B*07:02, HLA-B*08:01	1.317	0.304	–	–	–	22.61%
	−0.078	YVPWIGNGY	9	811–820	HLA-A*26:01	0.525	0.395	–	–	–	5.82%
	1.133	QYIPLFIDI	9	1,445–1,454	HLA-A*24:02	0.759	0.257	–	–	–	21.38%
HTL epitopes											
HMW1	−1	DYLQYVGNEAYGYYD	15	105–120	HLA-DRB1*04:01, HLA-DRB1*09:01	0.492	–	0.908	IL4-inducer	IL10-inducer	17.24%
	−0.967	RSLSNDFTIAHRPSD	15	825–840	HLA-DRB1*03:01	0.811	–	0.466	IL4-inducer	IL10-inducer	17.84%
HMW2	−0.54	ARTQFDNRVSLLSAR	15	608–623	HLA-DRB1*03:01	1.214	–	0.229	IL4-inducer	IL10-inducer	17.84%
	−0.527	QSQPAFLATQQSISK	15	1780–1795	HLA-DRB1*04:01, HLA-DRB1*01:01	0.61	–	0.296	IL4-inducer	IL10-inducer	22.06%
HMW3	0.68	TPIASRFTGVTPMAV	15	573–588	HLA-DRB1*01:01, HLA-DRB1*04:01, HLA-DRB1*07:01, HLA-DRB1*09:01	0.515	–	0.489	IL4-inducer	IL10-inducer	43.06%
p1	−0.5	PWTYRNTSFSSLPLT	15	68–83	HLA-DRB1*07:01, HLA-DRB1*09:01	1.602	–	0.337	IL4-inducer	IL10-inducer	24.01%
	−0.247	QRALIWAPRPWAAFR	15	1,155–1,170	HLA-DRB1*09:01, HLA-DRB1*15:01	0.497	–	1.721	IL4-inducer	IL10-inducer	24.18%
	−0.12	WAAFRGSWVNRLGRV	15	1,165–1,180	HLA-DRB1*01:01, HLA-DRB1*04:01, HLA-DRB1*07:01, HLA-DRB1*09:01, HLA-DRB1*15:01	0.453	–	0.768	IL4-inducer	IL10-inducer	56.72%
p65	−1.087	DPNQAYYAYVDPNAY	15	140–149	HLA-DRB1*04:01	0.527	–	1.246	IL4-inducer	IL10-inducer	11.21%

–: not detected.

### 3.2 Worldwide population coverage

**Figure 2:**
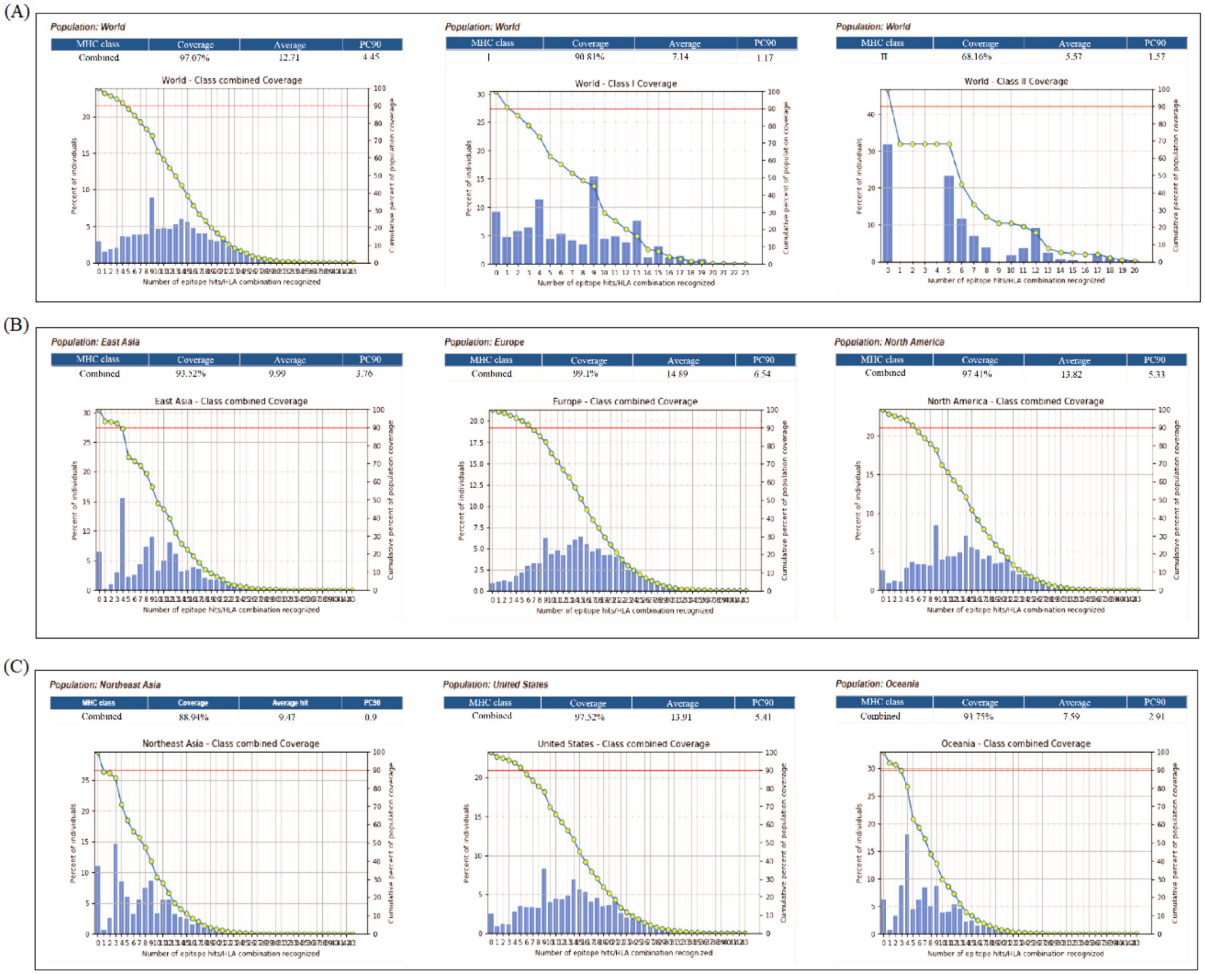
Global and regional population coverage analysis of the T-cell epitopes. **(A)** Worldwide population coverage for the combined CTL and HTL epitopes (97.07%), CTL epitopes alone (90.81%), and HTL epitopes alone (68.16%). **(B,C)** Regional coverage analysis demonstrates the vaccine’s high efficacy potential across diverse geographical regions, including North America, Europe, and East Asia. This broad coverage is attributed to the selection of epitopes with binding affinity for a wide array of HLA alleles, indicating the vaccine’s potential utility for a vast majority of the global population.

The population coverage is usually calculated for T-cell epitopes to ensure broad efficacy based on the variable distribution of HLA alleles worldwide. In this case, we assessed the population coverage of the selected CTL and HTL epitopes along with their corresponding binding alleles (Tables 1, 2). The global population coverage of the final multi-epitope vaccine was calculated to be 97.07%, which represents the combined coverage for the entire set of CTL and HTL epitopes, indicating that the vast majority of the world’s population is expected to respond to at least one epitope within the vaccine construct. Specifically, CTL epitopes had an average coverage of 90.81%, while HTL epitopes covered 68.16% globally (Figure 2). These epitopes showed binding affinity with various HLA alleles across regions such as the United States (97.52%), North America (97.41%), East Asia (93.52%), Northeast Asia (88.94%), Oceania (93.75%), and Europe (99.1%). Based on these findings, the vaccine targeting these epitopes is expected to be effective for a large portion of the global population (Figure 2).

### 3.3 Vaccine construct and fundamental properties

The vaccine includes 74 epitopes (21 linear B-lymphocyte epitopes, 34 CTLs and 19 HTLs), connected with AAY and GPGPG linkers (Tables 1, 2). A TLR4 agonist, the 50S ribosomal protein L7/L12 (17, 23), was linked to the CTL epitopes via an EAAAK linker to enhance immunogenicity, resulting in a vaccine consisting of 1,093 amino acids. This design enables the creation of a novel macro-molecule that combines the selected epitopes and a strong adjuvant, eliciting a robust immune response. Engineered to target both influenza A and *M. pneumoniae*, the vaccine leverages conserved epitopes identified in our analysis. The TLR4 agonist is expected to enhance both innate and adaptive immune responses, offering broad protection against these pathogens.

### 3.4 Physicochemical properties, immunological assessment and tertiary structure

**Figure 3:**
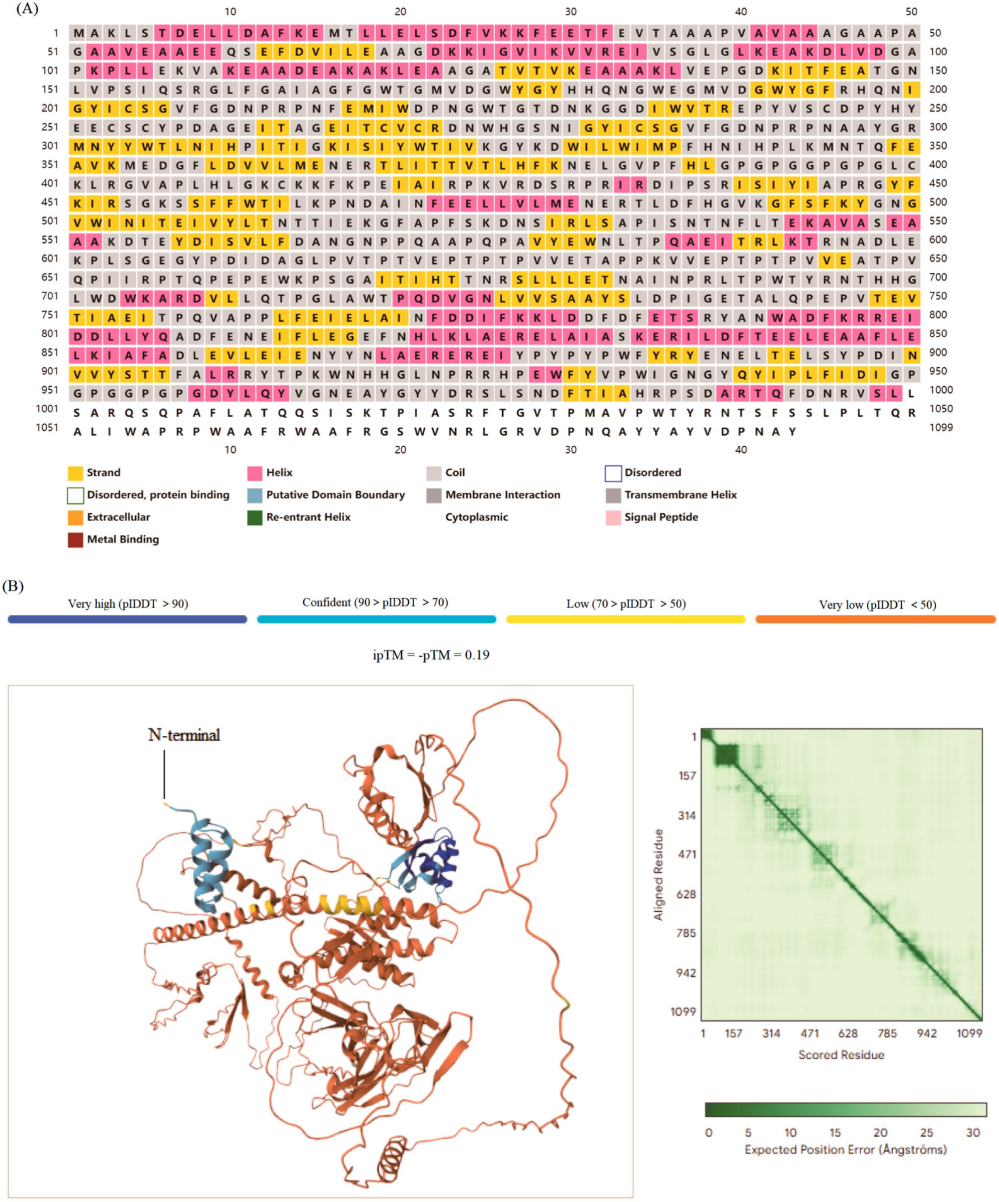
Secondary and tertiary structure prediction of the multi-epitope vaccine. **(A)** Predicted secondary structure showing the distribution of *α*-helices (red cylinders), *β*-strands (blue arrows), and random coils (gray lines). The high proportion of coils and helices is consistent with a soluble and stable protein. **(B)** The tertiary structure model generated by AlphaFold 3.0, color-coded by per-residue confidence (pLDDT) scores.

We assessed the physicochemical properties of the vaccine, revealing a molecular weight of 122.64 kDa, an antigenicity score of 0.75, and an immunogenicity score of 19.97, indicating strong antigenic and immunogenic potential. The vaccine’s theoretical isoelectric point (pI) was 5.14, with an instability index of 30.56, an aliphatic index of 81.52, and a GRAVY value of −0.3, showing a non-allergenic profile (Table 3). These results suggest high solubility and a low likelihood of allergic reactions. Secondary structural analysis predicted the presence of *α*-helix, random coils, and *β*-strands. PSIPRED estimated the structure to contain 18.76% (205/1093) α-helix, 13.17% (144/1093) β-strand, and 68.07% random coils, suggesting enhanced stability (Figure 3A). In addition, the 3D structure of the designed vaccine was generated by using AlphaFold 3.0 webserver and the median pLDDT (C-score) was predicted to be 72, which meant a high confidence (70 < pLDDT < 89) (Figure 3B).

**Table 3:** Comparative summary of the physicochemical properties of the multi-epitope vaccine.

Property	Value in this study	Reference value
Number of Amino Acids (aa)	1,093	–
Molecular Weight (kDa)	122.64	–
Theoretical pI	5.14	–
Instability Index (II)	30.56	<40 (stable)
Aliphatic Index	81.52	high value = high thermostability
GRAVY	−0.3	negative value = hydrophilic
Antigenicity (VaxiJen)	0.746	>0.4 (probable Antigen)
Immunogenicity (IEDB)	19.965	relative score
Allergenicity (AllerTOP)	Non-allergen	non-allergen

### 3.5 The optimization and verification of designed vaccine

**Figure 4:**
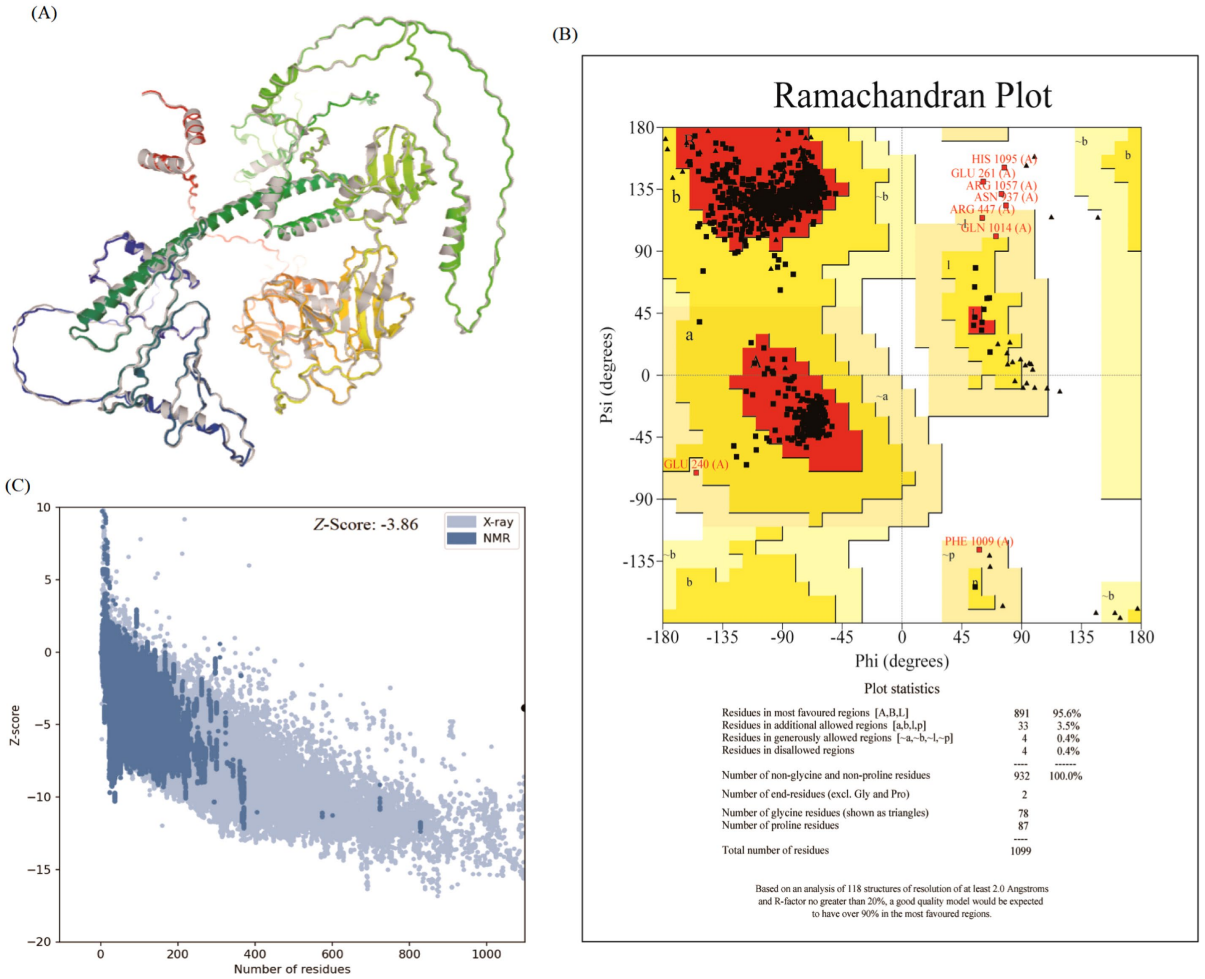
Validation and refinement of the vaccine’s 3D structural model. **(A)** The refined 3D structure of the vaccine candidate after processing through GalaxyRefine. **(B)** Ramachandran plot analysis of the refined model, showing that 95.6% of residues reside in the most favored regions, confirming excellent stereochemical quality and a near-native conformation. **(C)** ProSA-web *Z*-score of −3.86, which lies within the range of scores typically found for native proteins of similar size, validating the overall global model quality.

The 3D structure of the designed vaccine was further refined on the GalaxyRefine server (Figure 4A). The refined structure demonstrated excellent stereochemical quality, with Ramachandran plot analysis showing that 95.6% of residues were in favored regions, 3.5% in allowed regions, and only 0.4% in disallowed regions, indicating a near-native conformation (Figure 4B). The global structural quality was assessed by using the ProSA server with a *Z*-score of −3.86 (Figure 4C), consistent with X-ray and NMR data for over 85% of PDB structures. These results confirm the reliability of the vaccine model, with minimal structural deviations.

### 3.6 Disulfide engineering

**Figure 5:**
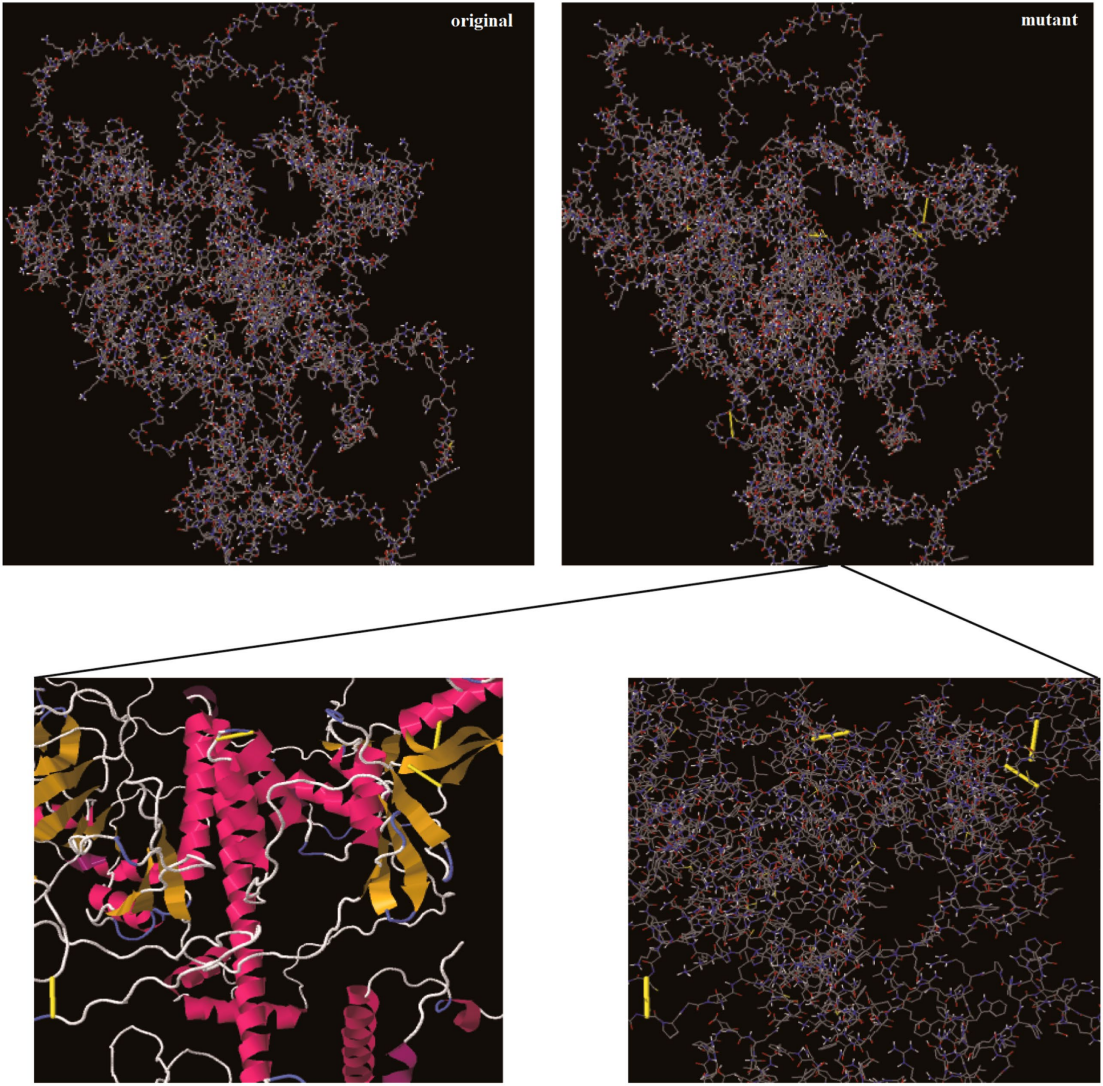
Disulfide engineering to enhance vaccine stability. Close-up view of the four residue pairs (Glu62-Ala132, Phe63-Ala113, Gln182-Gly184, Pro390-Gly394) selected for mutation to cysteine (shown as yellow sticks forming disulfide bonds). These engineered bonds were chosen based on an energy threshold of < 2.2 kcal/mol and are predicted to stabilize local loops and domains without distorting key epitopes, thereby increasing the structural rigidity of the vaccine candidate.

Disulfide bonds improve the stability of extracellular and secreted proteins by reducing their conformational entropy and increasing the free energy of denatured states, thereby stabilizing their folding form. During vaccine construction, stability is maintained through disulfide bonds between specific residues. The results showed that totally 21 potential residue pairs capable of forming disulfide bonds (Table 4). Four pairs such as Glu62 and Ala132, Phe63 and Ala113, Gln182 and Gly184, Pro390 and Gly394, were mutated based on a binding energy threshold of <2.2 kcal/mol (Table 4 and Figure 5).

**Table 4:** List of residue pairs in the constructed vaccine that are capable of forming disulfide bonds, along with their energy scores and chi 3 (dihedral angles).

Res1 AA	Res2 AA	Chi3	Energy
Met16	Glu20	125.44	3.31
**Glu62**	**Ala132**	**−93.68**	**1.92**
**Phe63**	**Ala113**	**107.93**	**2.18**
Val65	Leu105	94.8	4.49
Ala69	Thr126	86.33	4.22
Gly71	Gly124	80.8	3.57
Gly76	Ala123	121.25	3.96
Gly89	Glu92	103.71	2.32
Leu120	Ala125	114.4	5.05
Ala147	Pro214	−114	7.4
Ala165	Trp169	92.85	6.3
**Gln182**	**Gly184**	**120.5**	**1.44**
Asp229	Gly233	126.64	4.58
**Pro390**	**Gly394**	**93**	**2.08**
Pro431	Asp435	96.88	4.66
Pro446	Pro466	97.04	5.12
Lys451	Ser494	−99.42	8.29
Asn471	Glu474	115.16	3.46
Tyr896	Ile899	−110.58	2.89
Gly951	Gly954	120.93	5.47
Pro955	Asp959	97.07	4.41

The four pairs of disulfide bond residues selected for mutation were bolded to indicate.

Further analysis confirms that, of the four engineered disulfide bonds, only the Phe63-Ala113 pair (mutated to cysteines) lies within a single CTL epitope (NIHPITIGK), whose sequence is directly altered by the F63C substitution. Importantly, none of the other B-cell or T-cell epitopes are affected by our mutations. This demonstrates that our stabilization strategy successfully preserves the vast majority of the immunogenic architecture, including all key B-cell epitopes necessary for neutralizing antibody responses (Supplementary Figure S1 and Tables 1, 2). We therefore consider the alteration of a single, localized CTL epitope to be a favorable trade-off for achieving significant global stability. In addition, post-engineering immunogenicity and antigenicity (VaxiJen) predictions were comparable to the native construct, with scores changing negligibly from 19.965 to 19.778 and from 0.746 to 0.744, respectively. The mutated sequence showed non-allergenic and non-toxic characteristics.

### 3.7 Molecular docking

**Figure 6:**
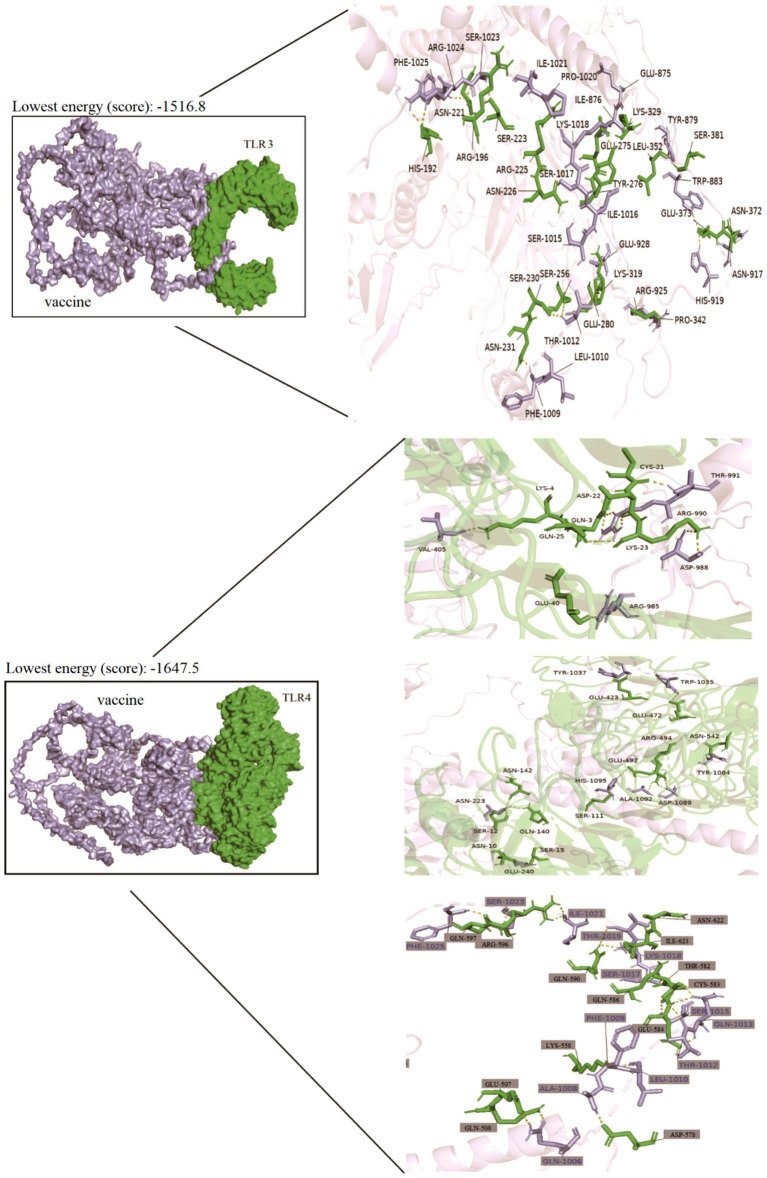
Molecular docking analysis of the vaccine with Toll-like receptors. The vaccine (shown in purple) is depicted in complex with TLR3 and TLR4 (shown in green). The models represent the lowest-energy docking poses. Yellow dashed lines indicate intermolecular hydrogen bonds between the vaccine and receptor residues. The extensive network of hydrogen bonds, particularly with key functional residues in the TLR active sites (e.g., the glycan-binding region of TLR3 and the LPS-binding pocket of TLR4), suggests high-affinity binding and provides a structural basis for the predicted robust immune activation.

The vaccine (ligand) was docked with the TLR3 and TLR4 receptors to predict interactions and binding affinity. The selected complex with the lowest binding energy was subjected to an analysis of binding interactions and an investigation of its involvement in active site residues. Regarding the vaccine-TLR3 complex, the number of hydrogen interactions present in the interaction plane was found to be 12. The residues that interacted with the hydrogen from the vaccine were Phe1025, Arg1024, Ser1023, Ser1017, Lys1018, Thr1012, Phe1009, Arg295, His919, Asn917. Furthermore, the associated TLR3 interacting residues were identified as His192, Asn221, Ser223, Asn226, Glu275, Ser256, Ser230, Glu280, Asn231, Pro342, Glu373, Asn372 (Figure 6).

Similarly, with regard to the vaccine-TLR4 complex, the number of hydrogen interactions present in the interaction plane was found to be 24. The residues that interacted with the hydrogen from the vaccine were Phe1025, Val405, Ser1023, Thr991, Asp988, Arg985, Asn223, Glu240, His1095, Ala1092, Asp1089, Tyr1084, Tyr1037, Trp1035, Gln1006, Ala1008, Leu1010, Thr1012, Ser1015, Thr1019. Furthermore, the associated TLR4 interacting residues were identified as Gln597, Lys4, Arg596, Cys21, Arg990, Glu40, Asn142, Ser12, Gln140, Asn10, Ser15, Ser111, Glu492, Arg494, Asn542, Glu423, Glu472, Glu507, Asp578, Lys558, Glu584, Cys583, Gln590 (Figure 6).

Several hydrogen-bonding residues identified in our vaccine-TLR complexes correspond to known functional sites critical for receptor activation. In the TLR3 complex, key interacting residues including His919 and Asn917 map to the conserved glycan-binding region, while Arg295 resides within the dimerization interface essential for signal transduction (24). Similarly, in the TLR4 complex, critical residues such as Asp988 and Arg985 participate in the conserved LPS-binding pocket, with Glu240 and Asn223 located in the MD-2 co-receptor dimerization interface (25). This strategic engagement with functionally established residues strongly supports the biological relevance of the predicted vaccine-receptor interactions and suggests a plausible mechanism for immune activation through native signaling pathways. This network of hydrogen bonds suggests a specific and energetically favorable binding mode, which is crucial for effective receptor recognition and subsequent immune activation.

### 3.8 Normal mode analysis

**Figure 7:**
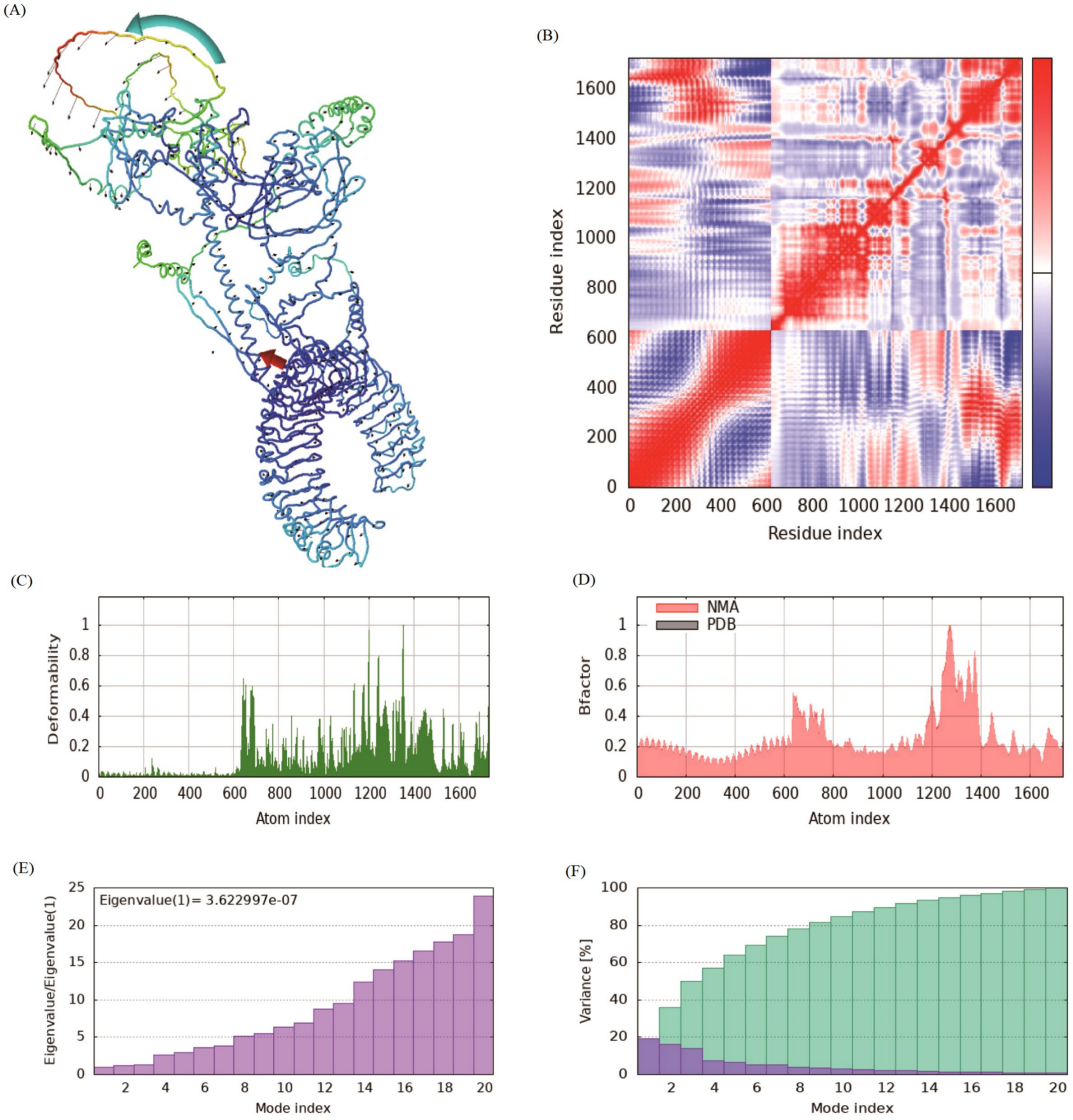
Normal mode analysis (NMA) of the vaccine-TLR3 complex. **(A)** Deformability map indicating regions of flexibility along the protein chain. **(B)** Covariance matrix depicting correlated (red), anti-correlated (blue), and uncorrelated (white) motions between residue pairs. The dominant red regions indicate coordinated movement, essential for functional binding. **(C)** B-factor values derived from NMA, showing good correlation with the flexibility observed in the deformability plot. **(D)** The eigenvalue associated with the slowest mode, reflecting the energy required for collective deformation. **(E,F)** The variance and cumulative variance for each normal mode, indicating that the complex’s essential motions are captured in the first few low-frequency modes. Collectively, these results validate the stability and plausible dynamics of the vaccine-TLR3 complex.

**Figure 8:**
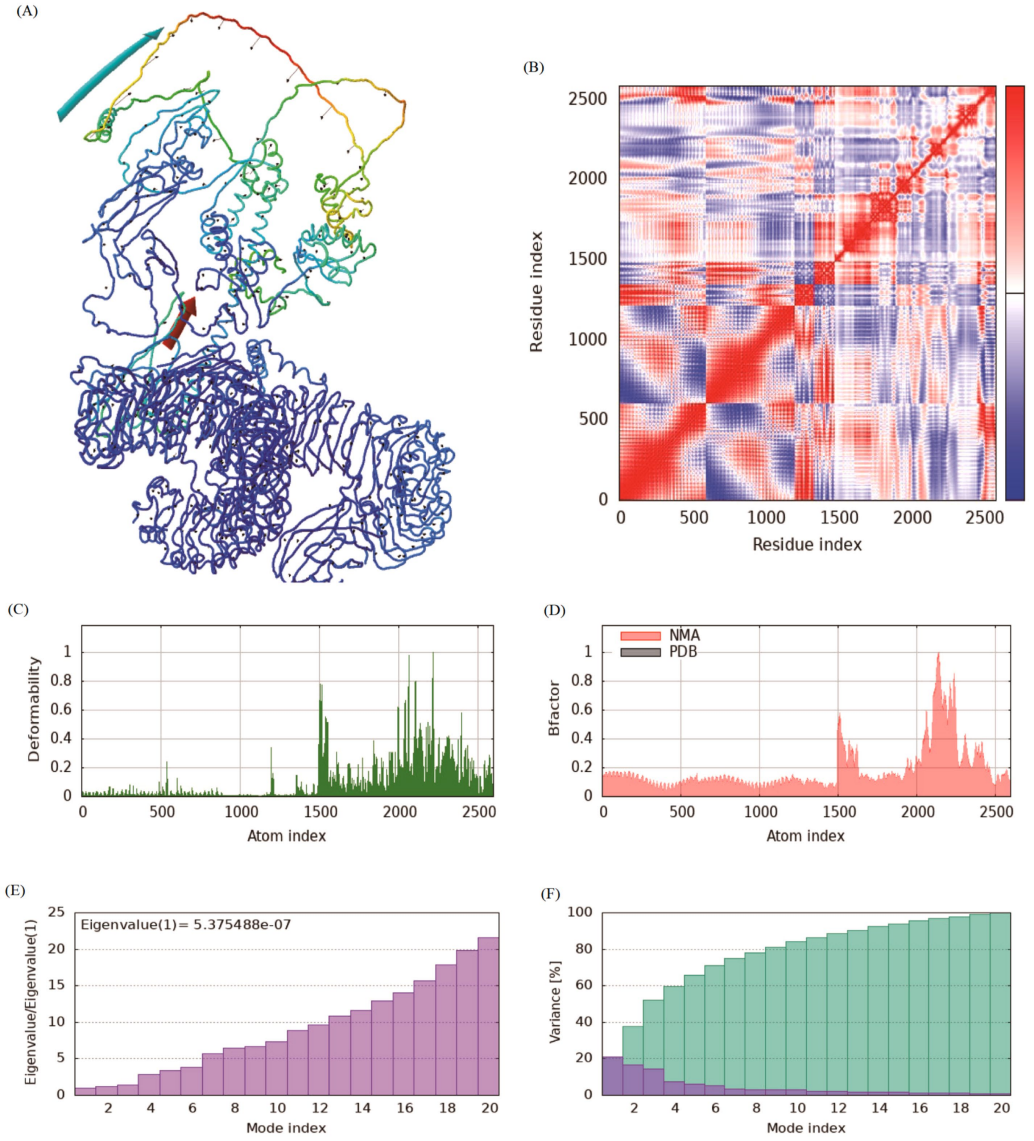
Normal mode analysis (NMA) of the vaccine-TLR4 complex. **(A)** Deformability map of the vaccine-TLR4 complex. **(B)** Covariance matrix showing strongly correlated motions (red) at the binding interface. **(C)** B-factor profile indicating stable regions with localized flexibility. **(D)** Eigenvalue of the slowest mode. **(E,F)** Variance and cumulative variance plots. The NMA results for the TLR4 complex corroborate the findings with TLR3, demonstrating low deformability and stable collective motions, which reinforces the prediction of a stable and specific interaction between the vaccine and TLR4.

Normal mode analysis based on iMODS is generally used to check the stability and motion of docking complexes. The slow kinetics of docking complexes and demonstrated their large amplitude conformational changes were analyzed using the normal mode analysis. The NMA was conducted for the designed multi-epitope vaccine after molecular docking with TLR3 and TLR4 receptors, respectively. The results showed that the receptor and ligand tended to cluster together (Figures 7A, 8A). The covariance map showed that the binding region covered sufficient red colors, indicating that the key region of the protein had coordinated amino acid movement and stable ligand binding (Figures 7B, 8B). The deformability built up the independent distortion of each residue portrayed by the method of chain hinges (Figures 7C, 8C). The designed vaccine protein exhibits generally low deformability when complexed with TLR4 and TLR3, indicating high structural stability. A few localized regions show peaks of flexibility, which may facilitate necessary conformational adjustments upon binding. This balance of overall rigidity and specific flexibility is favorable for a stable and effective immune response (Figures 7C, 8C). The B-factor analysis indicates the overall structural stability of the vaccine protein in complex with TLR3 and TLR4, with most regions exhibiting low values. A limited number of specific residues show higher B-factor peaks, suggesting localized flexibility in these areas. This pattern implies a generally rigid structure with targeted mobility, which is conducive to stable receptor binding (Figures 7D, 8D). Eigenvalue, which is a crucial parameter of a stable structure, must be high to have a stable complex. The eigenvalue of TLR3-vaccine and TLR4-vaccine complex were found to be 3.62E-7 and 5.38E-7, respectively, and the variance of each typical mode gradually decreased (Figures 7E,F, 8E,F). These rates are significantly higher for structural stability. The moderate eigenvalues indicated that the protein maintained its biological activity after binding and adapted to ligand binding.

### 3.9 Codon optimization and vaccine cloning

**Figure 9:**
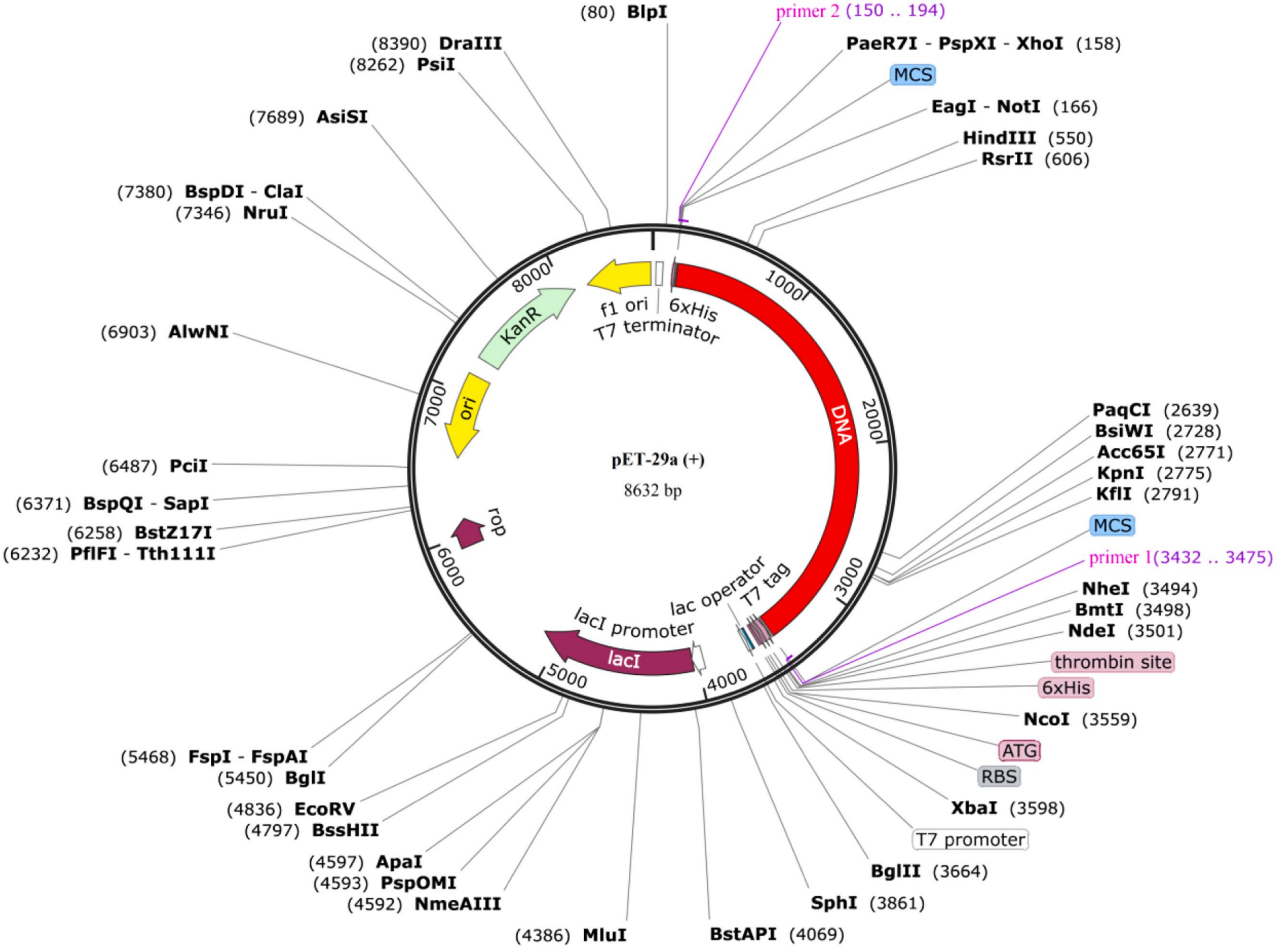
*In silico* cloning of the vaccine sequence into an expression vector. Map of the recombinant pET-29a(+) expression vector containing the codon-optimized vaccine insert (highlighted in red). The successful virtual cloning and the high Codon Adaptation Index (CAI = 1.0) and optimal GC content (53%) confirm the construct’s high potential for efficient recombinant expression in *E. coli* K12 systems.

To optimize vaccine expression, codon optimization was performed by using the JCat servers. The results showed that DNA length of the designed vaccine is 3,282 bp, with a stop codon added at the end. The CAI value (1) and GC content (53%) indicated strong gene expression potential in the model strain *E. coli* K12. The DNA sequence was then cloned into the pET-29a (+) vector using SnapGene v5.2.3 (Figure 9).

## 4 Discussion

Co-infections caused by the influenza A virus and *M. pneumoniae* present significant clinical challenges, particularly in immunocompromised individuals. While vaccines exist for certain strains of influenza A, there is no widely available vaccine for *M. pneumoniae*, highlighting the requirement of a dual vaccine capable of targeting both pathogens (15). A dual vaccine should integrate conserved epitopes from both pathogens, such as the HA and NA proteins (26) and p30 adhesion protein (20). This study presents a promising approach by designing a dual vaccine utilizing immunoinformatics strategies to predict and select highly immunogenic epitopes from both influenza A and *M. pneumoniae*. Due to the relatively high mutation rate of the viral genomes, we generated consistent sequences for the crucial antigens from different strains of influenza A and *M. pneumoniae*, respectively, and identified conserved and immune dominant epitopes from them. These sequences have broad spectrum, ensuring that this designed vaccine has sustained protection against viral infections. These epitopes were selected based on their antigenicity and immunogenicity scores to ensure their potential to stimulate a strong immune response. Epitope prediction, combined with their alignment with experimentally validated epitopes in the IEDB database, further substantiated their role in the vaccine design (27–32). The inclusion of 21 B-cell epitopes, 34 CTL and 19 HTL epitopes targeting influenza A and *M. pneumoniae* ensures a comprehensive immune response, offering both cellular and humoral immunity. The incorporation of a potent TLR3/TLR4 agonist enhances immune system activation, promoting both innate and adaptive immune responses, providing broad-spectrum immunity (33–35). Population coverage analysis demonstrated that the vaccine epitopes offer global coverage, with particularly high coverage in regions heavily affected by both influenza A and *M. pneumoniae*, such as North America, Europe, and East Asia (Figure 2). The widespread applicability of the vaccine suggests its potential to be highly effective across diverse global populations, potentially mitigating the impact of the co-infections (28–30). The physicochemical properties of the vaccine candidate were thoroughly evaluated, showing a favorable profile with a moderate molecular weight (Table 3). Firstly, the instability index of 30.56 indicates that the vaccine structure is relatively stable, which is beneficial for the integrity of production and storage processes (17, 36). Secondly, the theoretical pI is 5.14 and the GRAVY value is −0.30, indicating that it has moderate hydrophilicity and solubility in physiological environments, which facilitates effective interaction with immune cells (17). Finally, the protective antigen prediction score of 0.746 combined with the immunogenicity score of 19.965 further validated its potential to trigger potent immune responses, providing a theoretical basis for subsequent experiments (17). Strong antigenicity and immunogenicity scores suggest that the vaccine is stable and effective at inducing immune responses (37). Secondary structure analysis revealed a high content of *α*-helix and random coils, both associated with enhanced stability and solubility (Figure 3). These structural features are crucial for the practical application and production of the vaccine (38). The 3D structure of the vaccine was optimized using AlphaFold 3.0, which demonstrated excellent stereochemical quality and a nearly native conformation. The Ramachandran analysis of the refined 3D structure revealed that most of the residues were in favorable regions (Figure 4), confirming the structural reliability (37). The rationale for selecting TLR3 and TLR4 as molecular docking targets in this study is strongly supported by their well-defined roles in the innate immune response to the respective pathogens. TLR3 is a key pattern recognition receptor that specifically identifies dsRNA, a molecular signature generated during the replication cycle of influenza A viruses. This recognition triggers antiviral signaling pathways, making TLR3 a highly relevant target for investigating influenza A. Conversely, TLR4 is primarily responsible for recognizing bacterial membrane components, such as lipoproteins and other surface-associated molecular patterns. Despite the atypical cell wall of *M. pneumoniae*, it possesses membrane constituents that can engage TLR4, initiating a pro-inflammatory immune response. Thus, the inclusion of both TLR3 and TLR4 is justified by their distinct and critical immunological functions in sensing influenza A and *M. pneumoniae*, respectively, providing a solid foundation for our molecular docking analysis. In this case, molecular docking studies had been conducted, which modeled interactions between the vaccine and the TLR4/TLR3 receptors (34). The docking results for TLR4 (Figure 6) indicated a stable complex with an the lowest energy, suggesting strong binding affinity. At least 24 pairs of residues were involved in the hydrogen bonding interaction, supporting the hypothesis that the vaccine can activate immune responses through TLR4, a critical receptor for innate immunity (35). Similarly, the binding interface analysis revealed at least 12 hydrogen bonds between the vaccine and TLR3, with key residues stabilizing the interaction (Figure 6). These findings suggest that the vaccine may activate the TLR-mediated immune signaling pathway, providing an additional mechanism for immune system activation (33–35). In general, the designed vaccine demonstrates a strategic and comprehensive advancement over previous studies by integrating a rationally selected multi-antigen approach with enhanced structural and immunological properties. The target antigens such as HA and NA for influenza A, and p1, p65, HMW1-3 for *M. pneumoniae*, were specifically chosen based on their established critical roles in pathogenesis, serving as primary targets for neutralizing antibodies and key mediators of bacterial adhesion, respectively (12, 15). To ensure broad-spectrum efficacy, we employed consensus sequences derived from multiple circulating strains, thereby overcoming the limitations of single-strain designs. This integrated strategy resulted in epitopes with superior predicted immunogenicity and the capacity to induce IFN-*γ*, ultimately achieving markedly higher worldwide population coverage. Furthermore, the stability and structural reliability of our vaccine were significantly improved through disulfide engineering, yielding a refined model with exceptional stereochemical quality. Collectively, these elements distinguish our construct as a robust and broadly protective candidate against both pathogens.

The vaccine designed for influenza A and *M. pneumoniae* has many advantages compared to the associated vaccines. First, at present, there is a lack of vaccines designed to combine influenza A and *M. pneumoniae*. In most cases, they are the single vaccine with poor broad-spectrum efficacy. Second, in terms of antigen selection, Mahmood et al. (2021) (37) selected the antigenic proteins from a single strain (strain ATCC 29342/M129), while we chose 5 important structural proteins (HMW1-3, p1-adhesive protein, and p65) from different strains of *M. pneumoniae* as antigens and generated consensus sequences to ensure a broad antigen coverage. Besides, Samantaray et al. (2025) (38) also selected the single protein sequences of the pandemic (2009) IAV H1N1 strain for analysis, which lacked antigen broad-spectrum. In contrast, in the design of influenza vaccines, although Almalki et al. (2022) (39) used conserved sequences of a large number of strains for vaccine design, they only collected neuraminidase protein (NA) proteins from different H1N1 strains for vaccine design, which has limitations in its broad-spectrum. Second, the immunogenicity scores of 7 selected CTL epitopes by Mahmood et al. (2021) (37) were very low (0.02–0.38, average score: 0.121), while the CTL epitopes of *M. pneumoniae* in this study were predicted to have high immunogenicity (0.2–0.395, average score: 0.274). Third, most of the HTL epitopes selected by Mahmood et al. (2021) (37) were predicted to have no capacity of inducing the synthesis of IFN-γ, while the HTL epitopes of *M. pneumoniae* in this study were predicted to be able to induce IFN-γ production. Fourth, in terms of population coverage assessment, the MEV designed by Mahmood et al. (2021) (37) only had a 83.12% worldwide population coverage, while the vaccine designed in this study had a 97.07% worldwide population coverage for both influenza A and *M. pneumoniae*. In contrast, Samantaray et al. (2025) (38) did not make any population coverage analysis for the designed influenza vaccine. In this study, the disulfide bonds were added to ensure the stability and reliability of vaccine design (Figure 5), while the research by Mahmood et al. (2021) (37) and Almalki et al. (2022) (39) lacked the introduction of disulfide bonds. Finally, in the 3D structure evaluation of MEV, only 71% of the residues in the MEV designed by Mahmood et al. (2021) (37), 90% of the residues by Almalki et al. (2022) (39) and 90.1% by Samantaray et al. (2025) (38) were located in favored regions, however, 95.6% of the residues in this study for influenza A and *M. pneumoniae* were located in the favored regions, which was a higher proportion here than the previous studies. In summary, the multi-epitope vaccine designed here is significantly superior to that by previous studies (37, 39) in terms of immunogenicity, population coverage, physicochemical properties, and protein conformation.

A reasonable concern regarding the design of dual target vaccines is whether a single construct can effectively trigger immune responses against two different biological pathogens. However, the immunoinformatic driven approach suggests that the immune system can recognize and respond to conserved epitopes of both pathogens through a shared mechanism of antigen presentation and T/B cell activation. The selected epitopes have undergone rigorous screening and possess antigenicity, immunogenicity, non allergenicity, and broad HLA coverage, ensuring their ability to induce cellular and humoral immunity. In addition, the addition of potent TLR3/TLR4 agonist adjuvants can enhance innate immune activation, which is crucial for initiating adaptive responses against multiple pathogens. This strategy is supported by previous studies, which have shown that multi-epitope vaccines can provide protection against systemically unrelated pathogens by participating in common immune pathways (17). In addition, it may be feasible to study the combination strategies of different pathogens. Recently, Yu et al. (2024) (40) developed a *M. pneumoniae* genetic engineering vaccine with influenza virus strain as vector, and confirmed to have immunogenicity. Besides, the COVID-19 and influenza vaccine developed by Xing et al. (2024) (41) was proved to trigger a strong and balanced immune response against two pathogens, providing a feasible strategy for developing a multivalent vaccine against respiratory viruses. Therefore, although the biological characteristics of influenza A and *M. pneumoniae* are different, the conservation of selected epitopes and the universal mechanism of immune recognition support the feasibility of a single vaccine targeting these two organisms.

Despite the promising results, there are areas for optimization. The immunogenicity of HTL epitopes, while strong, may still be improved through modifications in epitope length or linker design to maximize helper T cell responses. Furthermore, while global population coverage is high, refining the epitope selection to account for regional variations in HLA allele frequencies could further enhance vaccine efficacy in underrepresented populations (42, 43). Nevertheless, predictions are *in silico* only and need validation, further experimental studies are still needed to substantiate the vaccine’s effectiveness in preventing infections. In the future, integrating deep learning-based epitope prediction and real-world pathogen surveillance data may further refine the design of dual vaccines with enhanced precision. Moreover, the inclusion of possible delivery strategies such as nanoparticle carriers, mucosal adjuvants could significantly improve antigen uptake and cross-protective efficacy. Continued *in vivo* validation and translational studies will be critical in transforming this computational framework into a viable immunization strategy for clinical usage (22).

## 5 Conclusion

In this study, we developed a dual vaccine targeting both influenza A and *M. pneumoniae* with immunoinformatics methods. The vaccine demonstrated strong antigenicity, favorable physicochemical properties, and extensive global population coverage. Molecular docking with TLR3 and TLR4 revealed stable binding interactions, suggesting effective immune activation. The approach in this study offered a solid framework for developing vaccines against multiple pathogens.

## Data Availability

The raw data supporting the conclusions of this article will be made available by the authors, without undue reservation. All mentioned data and codes used for the data analysis in this study are publicly available. The script used for consensus sequences construction is available at: https://github.com/xielisos567/Consensus.

## Footnotes

1
https://www.viprbrc.org/

2
https://www.ncbi.nlm.nih.gov/

3
https://www.ncbi.nlm.nih.gov/genomes/FLU/Database/nph-select.cgi?go=database

4
http://www.ddg-pharmfac.net/vaxijen/VaxiJen/VaxiJen.html

5
https://webs.iiitd.edu.in/raghava/toxinpred/index.html

6
http://tools.iedb.org/conservancy/

7
http://tools.iedb.org/immunogenicity/

8
https://www.iedb.org/

9
https://webs.iiitd.edu.in/raghava/abcpred/ABC_submission.html

10
https://web.expasy.org/protparam/

11
https://protein-sol.manchester.ac.uk

12
http://bioinf.cs.ucl.ac.uk/psipred/

13
https://alphafoldserver.com/

14
https://galaxy.seoklab.org/cgi-bin/submit.cgi?type=REFINE

15
https://www.pymol.org/

16
https://prosa.services.came.sbg.ac.at/prosa.php

17
https://saves.mbi.ucla.edu/

18
http://cptweb.cpt.wayne.edu/DbD2/

19
https://www.rcsb.org/

20
https://cluspro.bu.edu/home.php

21
http://imods.chaconlab.org/

22
http://www.jcat.de/
