# Supplementary figures and images for: Designing a broad-spectrum multi-epitope vaccine against influenza A and Mycoplasma pneumoniae: an immunoinformatics approach
Source: Front Public Health. 2026 Jan 21;14:1671035. doi: 10.3389/fpubh.2026.1671035 (PMC12868154; doi:10.3389/fpubh.2026.1671035)

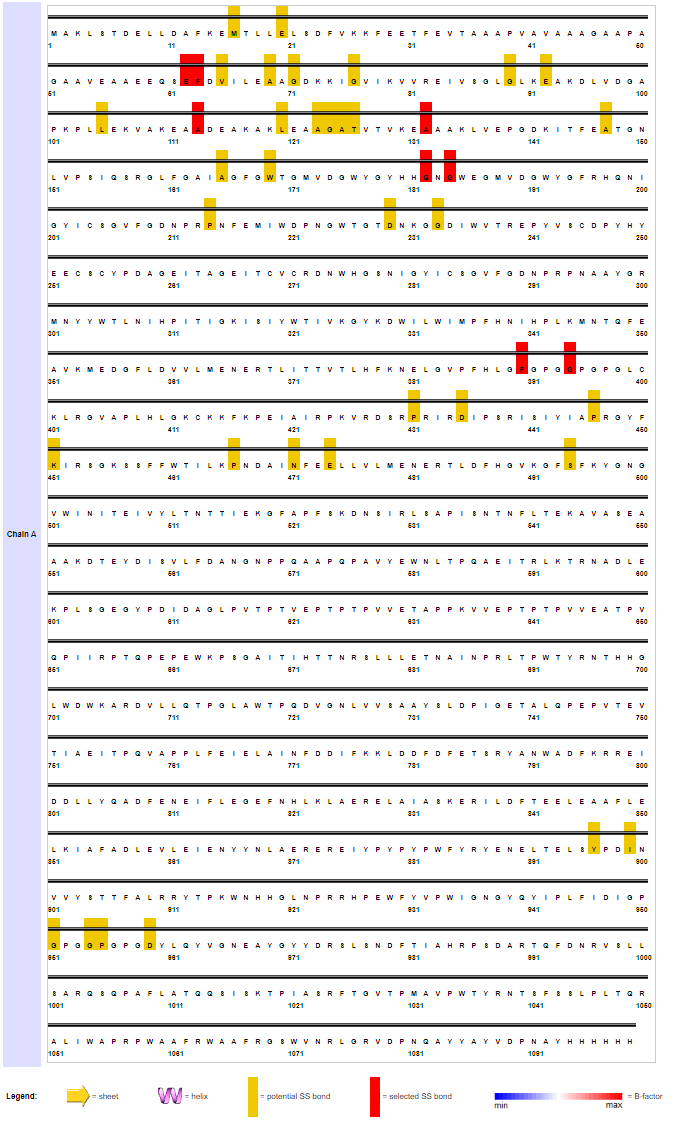

Supplement: Supplementary file 1 [file Image_1.png]
